# A process of rumour scotching on finite populations

**DOI:** 10.1098/rsos.150240

**Published:** 2015-09-16

**Authors:** Guilherme Ferraz de Arruda, Elcio Lebensztayn, Francisco A. Rodrigues, Pablo Martín Rodríguez

**Affiliations:** 1 Departamento de Matemática Aplicada e Estatística, Instituto de Ciências Matemáticas e de Computação, Universidade de São Paulo - Campus de São Carlos, Caixa Postal 668, São Carlos, São Paulo 13560-970, Brazil; 2 Instituto de Matemática, Estatística e Computação Científica, Universidade Estadual de Campinas - UNICAMP, Rua Sérgio Buarque de Holanda 651, Campinas, São Paulo 13083-859, Brazil

**Keywords:** rumour process, asymptotic behaviour, density-dependent Markov Chain, Monte Carlo simulation, epidemic model, stochastic model

## Abstract

Rumour spreading is a ubiquitous phenomenon in social and technological networks. Traditional models consider that the rumour is propagated by pairwise interactions between spreaders and ignorants. Only spreaders are active and may become stiflers after contacting spreaders or stiflers. Here we propose a competition-like model in which spreaders try to transmit an information, while stiflers are also active and try to scotch it. We study the influence of transmission/scotching rates and initial conditions on the qualitative behaviour of the process. An analytical treatment based on the theory of convergence of density-dependent Markov chains is developed to analyse how the final proportion of ignorants behaves asymptotically in a finite homogeneously mixing population. We perform Monte Carlo simulations in random graphs and scale-free networks and verify that the results obtained for homogeneously mixing populations can be approximated for random graphs, but are not suitable for scale-free networks. Furthermore, regarding the process on a heterogeneous mixing population, we obtain a set of differential equations that describes the time evolution of the probability that an individual is in each state. Our model can also be applied for studying systems in which informed agents try to stop the rumour propagation, or for describing related susceptible–infected–recovered systems. In addition, our results can be considered to develop optimal information dissemination strategies and approaches to control rumour propagation.

## 1. Introduction

Spreading phenomena is ubiquitous in nature and technology [1]. Diseases propagate from person to person [2], viruses contaminate computers worldwide and innovation spreads from place to place [3,4]. In the last decades, the analysis of the phenomenon of information transmission from a mathematical and physical point of view has attracted the attention of many researchers [1,5–10]. The expression ‘information transmission’ is often used to refer to the spreading of news or rumours in a population or the diffusion of a virus through the Internet. These random phenomena have similar properties and are often modelled by mathematical models [5–7].

In this paper, we propose and analyse a process of rumour scotching on finite populations. An interacting particle system is considered to represent the spreading of the rumour by agents on a given graph, representing a finite population of size *n*. We assume that each agent, or node of the graph, may be in any of the three states belonging to the set {0,1,2}, where 0 stands for ignorant, 1 for spreader and 2 for stifler. Finally, the model is formulated by considering that a spreader tells the rumour to any of its (nearest) ignorant neighbours at rate λ and that a spreader becomes a stifler owing to the action of its (nearest neighbour) stifler nodes at rate *α*.

When the considered graph is the complete graph, representing a finite homogeneously mixing population, we obtain limit theorems regarding the proportion of ignorants at the end of the process. That is, when there are not more spreaders in the population. In addition, we study the model in random graphs and scale-free networks through Monte Carlo simulations. The computational approach allows us to verify that the results obtained for homogeneously mixing populations can be approximated for random graphs, but are not suitable for scale-free networks. Finally, we provide an analytical framework to understand the behaviour of the process on a heterogeneous mixing population. More precisely, we obtain a set of differential equations describing the time evolution of the probability that an individual is in each state. We show that there is a remarkable matching between these analytical results and those obtained from computer simulations.

We point out that a removal mechanism different from the one considered in the usual models is considered here. We assume that stifler nodes can scotch the rumour propagation. Our model is inspired by the stochastic process discussed in [11]. In such work, the author assumes that the propagation of a rumour starts from one individual, who after an exponential time learns that the rumour is false and then starts to scotch the propagation by the individuals previously informed. When the population is homogeneously mixed, Bordenave [11] showed that the scaling limit of this process is the well-known birth-and-assassination process, introduced in the probabilistic literature by Aldous & Krebs [12] as a variant of the branching process [13]. In order to introduce a more realistic model we consider two modifications. We suppose that each stifler tries to stop the rumour diffusion by all the spreaders that he/she meets along the way. It is assumed that the rumour starts with general initial conditions.

Our model can be applied to describe the spreading of information through social networks. In this case, a person propagates a piece of information to another one and then possibly becomes a stifler. That event may occur if, for instance, such person discovers that the piece of information is false and then tries to scotch the spreading. The same dynamics can model the spreading of data in a network. A computer can try to scotch the diffusion of a file after discovering that it contains a virus.

These dynamics are related to the well-known Williams–Bjerknes (WB) tumour growth model [14], which is studied on infinite regular graphs like hypercubic lattices and trees (see for instance [15–17]). The same model on the complete graph is studied by Kortchemski [18] in the context of a predator–prey susceptible–infected–recovered (SIR) model. As a description of a rumour dynamic on graphs with a finite number of vertices, including random graphs and scale-free networks, this model has not been addressed yet. In this way, here we apply the theory of convergence of density-dependent Markov chains and use computational simulations to study the asymptotic behaviour of rumour scotching on finite populations.

Our results can contribute to the analysis of optimal information dissemination strategies [19] as well as the statistical inference of rumour processes [20]. In addition, given the competition-like structure of the process, it may be applied as a toy model of marketing policies. In such a situation, the first spreader may represent the first individual to try a new product and his/her neighbours can imitate him/her at rate λ. On the other hand, stiflers may represent individuals who know that the product is low quality and therefore, they can persuade other users to dismiss the product at rate *α*. We refer the reader to [3,4], for a review of related models and results in this direction.

## 2. Previous works on rumour spreading

The most popular models to describe the spreading of news or rumours are based on the stochastic or deterministic version of the classical SIR, SIS (susceptible–infected–susceptible) and SI (susceptible–infected) epidemic models [1,21]. In these models, it is assumed that an infection (or information) spreads through a population subdivided into three classes (or compartments), i.e. susceptible, infective and removed individuals. In the case of rumour dynamics, these states are referred as ignorant, spreader and stifler, respectively.

The first stochastic rumour models are due to Daley & Kendall (DK) [22,23] and to Maki & Thompson (MT) [24]. Both models were proposed to describe the diffusion of a rumour through a closed homogeneously mixing population of size *n*, i.e. a population described by a complete graph. Initially, it is assumed that there is one spreader and *n*−1 are in the ignorant state. The evolution of the DK rumour model can be described by using a continuous time Markov chain, denoting the number of nodes in the ignorant, spreader and stifler states at time *t* by *X*(*t*), *Y* (*t*) and *Z*(*t*), respectively. Thus, the stochastic process {(*X*(*t*),*Y* (*t*))}_*t*≥0_ is described by the Markov chain with transitions and corresponding rates given by

$$\begin{array}{cc} \mathrm{transition} & \mathrm{rate}\\ \left(-1,1\right) & XY,\\ \left(0,-2\right) & \left(\begin{array}{c} Y\\ 2 \end{array}\right),\\ \left(0,-1\right) & Y(n-X-Y). \end{array}$$

This means that if the process is in state (*X*,*Y*) at time *t*, then the probability that it will be in state (*X*−1,*Y* +1) at time *t*+*h* is *XY* *h*+*o*(*h*), where *o*(*h*) is a function such that $\lim_{h\to 0}o(h)/h=0$. In this model, it is assumed that individuals interact by pairwise contacts and the three possible transitions correspond to spreader–ignorant, spreader–spreader and spreader–stifler interactions. In the first transition, the spreader tells the rumour to an ignorant, who becomes a spreader. The two other transitions indicate the transformation of the spreader(s) into stifler(s) because of its contact with a subject who already knew the rumour.

MT formulated a simplification of the DK model by considering that the rumour is propagated by directed contact between the spreaders and other individuals. In addition, when a spreader *i* contacts another spreader *j*, only *i* becomes a stifler. Thus, in this case, the continuous-time Markov chain to be considered is the stochastic process {(*X*(*t*),*Y* (*t*))}_*t*≥0_ that evolves according to the following transitions and rates:

$$\begin{array}{cc} \mathrm{transition} & \mathrm{rate}\\ \left(-1,1\right) & XY,\\ \left(0,-1\right) & Y(n-X). \end{array}$$

The first references about these models [22–24] are the most cited works about stochastic rumour processes in homogeneously mixing populations and have triggered numerous significant research in this area. Basically, generalizations of these models can be obtained in two different ways. The first generalizations are related to the dynamic of the spreading process and the second ones to the structure of the population. In the former, there are many rigorous results involving the analysis of the remaining proportion of ignorant individuals when there are no more spreaders on the population [25,26]. Note that this is one way to measure the range of the rumour. After the first rigorous results, namely limit theorems for this fraction of ignorant individuals [25,26], many authors introduced modifications in the dynamic of the basic models in order to make them more realistic. Recent papers have suggested generalizations that allow various contact interactions, the possibility of forgetting the rumour [27], long-memory spreaders [28] or a new class of uninterested individuals [29]. Related processes can be found for instance in [30,31]. However, all these models maintain the assumption that the population is homogeneously mixing.

On the other hand, recent results have analysed how the topology of the considered population affects the diffusion process. In this direction, Coletti *et al.* [32] studied a rumour process when the population is represented by the *d*-dimensional hypercubic lattice and Comets *et al.* [33] modelled the transmission of information of a message on the Erdos–Rényi (ER) random graph. Related studies can be found in [34–38] and references therein. In the previous works, authors dealt with different probabilistic techniques to get the desired results. Such techniques allow extending our understanding of a rumour process in a more structured population, namely, represented by lattices and random graphs. Unfortunately, when one deals with the analysis of these dynamics in real-world networks, such as online social networks or the Internet [39], whose topology is very heterogeneous, it is difficult to apply the same mathematical arguments and a different approach is required. In this direction, general rumour models are studied in [40,41] where the population is represented by a random graph or a complex network and important results are obtained by means of approximations of the original process and computational simulations.

## 3. Homogeneously mixing populations

The model proposed here assumes that spreaders propagate the rumour to their direct neighbours, as in the original MT model [24]. However, differently from this model, stifler nodes try to scotch the rumour propagation. Indeed, we assume that a spreader tells the rumour to an ignorant at rate λ and a spreader becomes a stifler at rate *α* owing to the action of a stifler.

Let us formalize the stochastic process of interest. Consider a population of fixed size *n*. As usual, we denote the number of nodes in the ignorant, spreader and stifler state at time *t* by *X*^*n*^(*t*), *Y* ^*n*^(*t*) and *Z*^*n*^(*t*), respectively. We assume that $x_{0}^{n}$, $y_{0}^{n}$ and $z_{0}^{n}$ are the respective initial proportions of these individuals in the population and suppose that the following limits exist:

3.1 $$\begin{array}{rl} x_{0} & :=\lim_{n\to \infty }x_{0}^{n}>0,\\ y_{0} & :=\lim_{n\to \infty }y_{0}^{n},\\ \;\mathrm{and}\;\;z_{0} & :=\lim_{n\to \infty }z_{0}^{n}>0. \end{array}\}$$

Our rumour model is the continuous-time Markov chain *V* ^(*n*)^(*t*)={(*X*^*n*^(*t*),*Y* ^*n*^(*t*))}_*t*≥0_ with transitions and rates are given by

$$\begin{array}{cc} \mathrm{transition} & \mathrm{rate}\\ \left(-1,1\right) & \lambda XY,\\ \left(0,-1\right) & \alpha Y(n-X-Y). \end{array}$$

This means that if the process is in state (*X*,*Y*) at time *t* then the probabilities that it will be in states (*X*−1,*Y* +1) or (*X*,*Y* −1) at time *t*+*h* are, respectively, λ*XY* *h*+*o*(*h*) and *αY* (*n*−*X*−*Y*)*h*+*o*(*h*). Note that while the first transition corresponds to an interaction between a spreader and an ignorant, the second one represents the interaction between a stifler and a spreader. When *n* goes to infinity, the entire trajectories of this Markov chain, rescaled by *n*, have as a limit the set of differential equations given by

3.2 $$\begin{array}{rl} x^{\prime }(t) & =-\lambda x(t)y(t),\\ y^{\prime }(t) & =\lambda x(t)y(t)-\alpha y(t)z(t),\\ z^{\prime }(t) & =\alpha y(t)z(t)\\ \;\text{and}\;\;x(0) & =x_{0},\;y(0)=y_{0},\;z(0)=z_{0}. \end{array}\}$$

**Figure 1.**
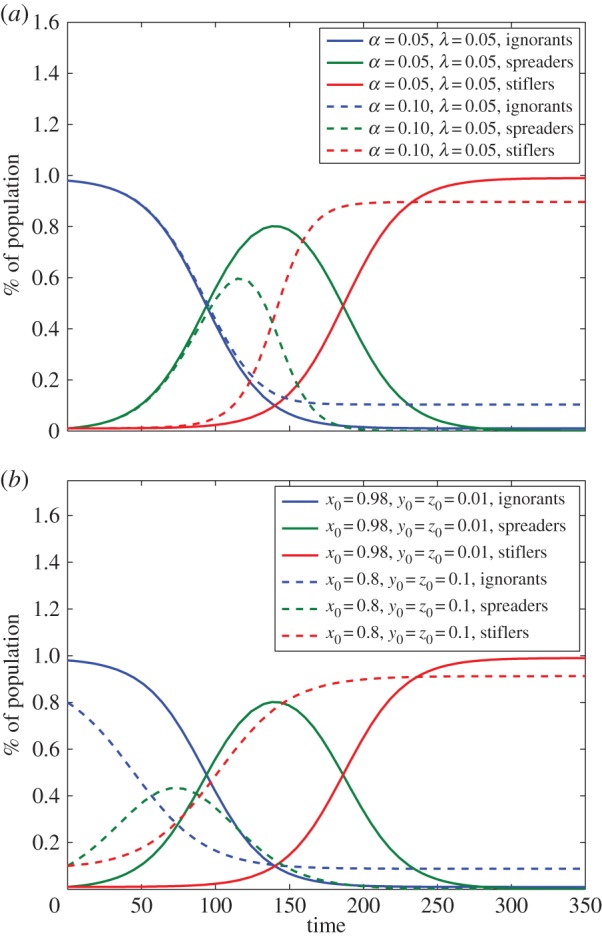
Time evolution of the rumour model (equation (3.2)) according to (*a*) the variation of the parameters *α* and λ for the fixed initial condition *x*_0_=0.98,*y*_0_= *z*_0_=0.01 and (*b*) the variation of the initial condition for the fixed parameters *α*=0.05,λ=0.05.

The solutions rely on the initial conditions, as the stifler class is an absorbing state. Figure 1 shows this dependency. In figure 1*a*, the initial conditions are fixed and two parameters *α* and λ are evaluated, showing that an increase in the values of *α* reduces the maximum fraction of spreader nodes. In figure 1*b*, the rates are fixed and the initial conditions are varied, which shows that the time evolution of the system changes, evidencing the dependency on the initial conditions.

**Figure 2.**
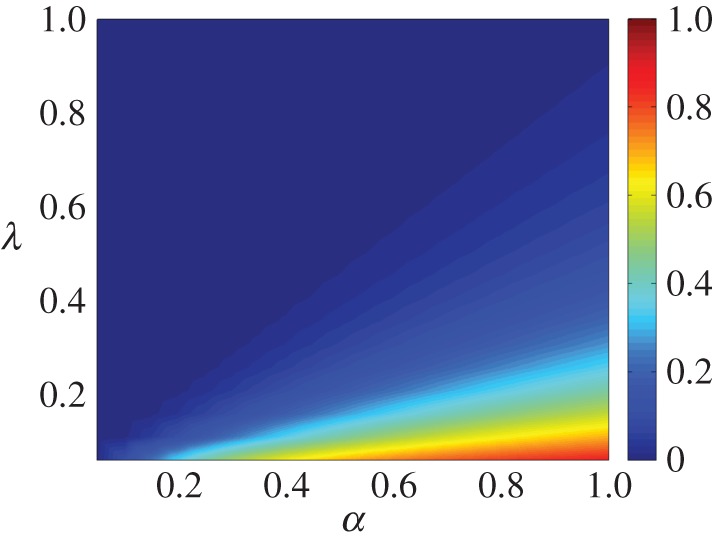
Fraction of ignorant individuals for the theoretical model, obtained by the numerical evaluation of the system of equations (3.2) for *x*_0_=0.98, *y*_0_=0.01 and *z*_0_=0.01.

We solved the system of equations (3.2) numerically for every pair of parameters, λ and *α*, each one starting from 0.05 and incrementing them with steps of 0.05 until reaching the unity. Figure 2*a* presents the results in terms of the fraction of ignorants at the end of the process. The higher the probability *α*, the higher the fraction of the ignorants for low values of λ. On the other hand, the fraction of ignorants is lower when the parameter λ is increased, even when *α*≈1.

The analysis of equations (3.2) allows us to obtain some information about the remaining proportion of ignorants at the end of the process. However, this procedure refers to the limit of the process and it does not say anything about the relation between such value and the size of the population. In order to study such relation, we consider the theory of density-dependent Markov chains, from which we can obtain not only information of the remaining proportion of ignorants, but also acquire a better understanding of the magnitude of the random fluctuations around this limiting value. This approach has already been used for rumour models, see for instance [28,29]. In the rest of the paper, we denote the ratio *α*/λ by *ρ*.

Let $\tau^{\left(n\right)}=\inf\{t:Y^{n}(t)=0\}$ be the absorption time of the process. More specifically, *τ*^(*n*)^ is the first time at which the number of spreaders in the population vanishes. Our purpose is to study the behaviour of the random variable *X*^*n*^(*τ*^(*n*)^)/*n*, for *n* large enough, by stating a weak law of large numbers and a central limit theorem.

The main idea is to define, by means of a random time change, a new process ${\left\{{\tilde{V}}^{\left(n\right)}(t)\right\}}_{t\geq 0}$, with the same transitions as {*V* ^(*n*)^(*t*)}_*t*≥0_, so that they terminate at the same point. The transformation is done in such a way that ${\left\{{\tilde{V}}^{\left(n\right)}(t)\right\}}_{t\geq 0}$ is a density-dependent Markov chain for which we can apply well-known convergence results (see for instance [42–44]).

The first step in this direction is to define

$$\theta^{n}(t)=\int_{0}^{t}Y^{n}(s)\;ds,$$

for 0≤*t*≤*τ*^(*n*)^. Notice that *θ*^*n*^ is a strictly increasing, continuous and piecewise linear function. In this way, we can define its inverse by

3.3 $$\Gamma^{n}(s)=\inf\{t:\theta^{n}(t)>s\},$$

for $0\leq s\leq \int_{0}^{\infty }Y^{n}(u)\;du$. Then it is not difficult to see that the process defined as

3.4 $${\tilde{V}}^{n}(t):=V^{n}(\Gamma^{n}(t))$$

has the same transitions as {*V* ^*n*^(*t*)}_*t*≥0_. As a consequence, if we define ${\tilde{\tau }}^{n}=\inf\{t:{\tilde{Y}}^{n}(t)=0\}$ we get that $V^{n}(\tau^{n})={\tilde{V}}^{n}({\tilde{\tau }}^{n})$. This implies that it is enough to study ${\tilde{X}}^{n}({\tilde{\tau }}^{\left(n\right)})/n$. The gain of the previous comparison relies on the fact that ${\left\{{\tilde{V}}^{n}(t)\right\}}_{t\geq 0}$ is a continuous-time Markov chain with initial state $\left(x_{0}^{n}n,y_{0}^{n}n\right)$ and transitions and rates given by

$$\begin{array}{cc} \mathrm{transition} & \mathrm{rate}\\ \ell_{0}=(-1,1) & \lambda X,\\ \ell_{1}=(0,-1) & \alpha (n-X-Y). \end{array}$$

In particular, the rates of the process can be written as

$$n\left[\beta_{l_{i}}\left(\frac{\tilde{X}}{n},\frac{\tilde{Y}}{n}\right)\right],$$

where *β*_ℓ_0__(*x*,*y*)=λ*x* and *β*_ℓ_1__(*x*,*y*)=*α*(1−*x*−*y*). Processes defined as above are called *density dependent* as the rates depend on the density of the process (i.e. normed by *n*). Then ${\left\{{\tilde{V}}^{n}(t)\right\}}_{t\geq 0}$ is a density-dependent Markov chain with possible transitions in the set {ℓ_0_,ℓ_1_}. By applying convergence results of [44], we obtain an approximation of this process, as the population size becomes larger, by a system of differential equations. Similar arguments have been applied for stochastic rumour and epidemic models [28,29,45] and we include them for the sake of completeness. We use the notation used in [44] except for the Gaussian process that we would rather denote by $V=(V_{x},V_{y})$. Here *φ*(*x*,*y*)=*y*, and

$$\tau_{\infty }=\inf\{t:y(t)\leq 0\}=-\frac{1}{\lambda }\log\left(\frac{x_{\infty }}{x_{0}}\right),$$

where $X_{\infty }$ represents the limiting fraction of ignorant individuals of the process, which is defined later. It is known that the limit behaviour of the density-dependent Markov chain ${\left\{{\tilde{V}}^{n}(t)\right\}}_{t\geq 0}$ can be determined by the drift function *F*(*x*,*y*)=*l*_0_*β*_0_(*x*,*y*)+*l*_1_*β*_1_(*x*,*y*).

In other words,

3.5 F(x,y)=(−λx,(λ+α)x+αy−α)

and the limiting system of ordinary differential equations is given by

3.6 $$\begin{array}{rl} x^{\prime }(t) & =-\lambda x(t),\\ y^{\prime }(t) & =(\lambda +\alpha )x(t)+\alpha y(t)-\alpha \\ \;\text{and}\;\;x(0) & =x_{0},y(0)=y_{0}. \end{array}\}$$

The solution of (3.6) is

3.7 $$\begin{array}{rl} x(t) & =x_{0}\exp(-\lambda t)\\ \;\text{and}\;\;y(t) & =f(x(t)), \end{array}\}$$

where $f:(0,x_{0}]\to R$ is given by

3.8 $$f(x)=1-(1-x_{0}-y_{0}){\left(\frac{x_{0}}{x}\right)}^{\rho }-x.$$

**Figure 3.**
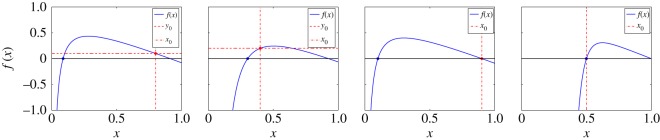
Four different cases for the function *f*(*x*) given by equation (3.8). (*a*) *ρ*<*x*_0_/*z*_0_ and *y*_0_>0, (*b*) *ρ*>*x*_0_/*z*_0_ and *y*_0_>0, (*c*) *ρ*<*x*_0_/*z*_0_ and *y*_0_=0 and (*d*) *ρ*>*x*_0_/*z*_0_ and *y*_0_=0.

Figure 3 shows the behaviour of *f*(*x*) for four possible relations between *ρ* and the initial conditions.

According to theorem 11.2.1 of [44] we have that, on a suitable probability space, ${\tilde{V}}^{n}(t)/n$ converges to (*x*(*t*),*y*(*t*)) given by (3.7), almost surely uniformly on bounded time intervals. Then the following results can be obtained as a consequence of theorem 11.4.1 of [44].

### 3.1 Law of large numbers

If $x_{\infty }$ denotes the root of *f*(*x*)=0 in (0,*x*_0_], then

3.9 $$\lim_{n\to \infty }\frac{X^{n}(\tau^{n})}{n}=x_{\infty }$$

in probability. This means that, for *n* large enough, it is a high probability the process dies out leaving approximately a proportion $x_{\infty }$ of remaining ignorant nodes of the population. In order to prove the limit of equation (3.9), note that *y*_0_>0 and

3.10 $$\nabla \varphi (v(\tau_{\infty }))\cdot F(v(\tau_{\infty }))=y^{\prime }(\tau_{\infty })=(\lambda +\alpha )x_{\infty }-\alpha <0$$

imply that $y(\tau_{\infty }-\varepsilon )>0$ and $y(\tau_{\infty }+\varepsilon )<0$ for $0<\varepsilon <\tau_{\infty }$. Therefore, the almost surely convergence of ${\tilde{Y}}^{n}(t)/n$ to *y*(*t*) uniformly on bounded intervals implies that

3.11 $$\lim_{n\to \infty }{\tilde{\tau }}^{\left(n\right)}=\tau_{\infty }\;a.s.$$

When *y*_0_=0 and *x*_0_>*ρz*_0_, this result is also valid because *y*′(0)>0 and (3.10) still holds. On the other hand, if *y*_0_=0 and *x*_0_≤*ρz*_0_, then *y*(*t*)<0 for all *t*>0, and again the almost sure convergence of ${\tilde{Y}}^{n}(t)/n$ to *y*(*t*) uniformly on bounded intervals yields that $\lim_{n\to \infty }{\tilde{\tau }}^{\left(n\right)}=0=\tau_{\infty }$ almost surely. Therefore, as ${\tilde{X}}^{n}(t)/n$ converges to *x*(*t*) almost surely, we obtain the law of large numbers from (3.11) and the fact that $X^{\left(n\right)}(\tau^{\left(n\right)})={\tilde{X}}^{\left(n\right)}({\tilde{\tau }}^{\left(n\right)})$.

### 3.2 Central limit theorem

Furthermore, we can describe the distribution of the random fluctuations around the limiting value $x_{\infty }$. More precisely, by assuming that *y*_0_>0, or that *y*_0_=0 and *ρ*<*x*_0_/*z*_0_, we obtain the following central limit theorem:

3.12 $$\sqrt{n}\left[\frac{X^{n}(\tau^{\left(n\right)})}{n}-x_{\infty }\right]\;\Rightarrow \;N(0,\sigma^{2})\;\mathrm{as}\;n\to \infty ,$$

where ⇒ denotes convergence in distribution and $N(0,\sigma^{2})$ is the Gaussian distribution with mean zero and variance *σ*^2^:=*σ*^2^(*α*,λ,*x*_0_,*y*_0_,*z*_0_) given by

3.13 $$\frac{x_{\infty }z_{\infty }[x_{0}x_{\infty }(1-z_{0}-x_{\infty })+z_{0}\rho^{2}z_{\infty }(x_{0}-x_{\infty })]}{x_{0}z_{0}{\left[\rho -x_{\infty }(\rho +1)\right]}^{2}},$$

where $z_{\infty }:=1-x_{\infty }$. Indeed, from theorem 11.4.1 of [44] we have that if, *y*_0_>0 or *y*_0_=0 and *x*_0_ > *ρz*_0_, then

$$\sqrt{n}(n^{-1}{\tilde{X}}^{n}({\tilde{\tau }}^{\left(n\right)})-x_{\infty })$$

converges in distribution as n→∞ to

3.14 $$V_{x}(\tau_{\infty })+\frac{x_{\infty }}{(1+\delta )x_{\infty }-\delta }V_{y}(\tau_{\infty }).$$

The resulting normal distribution has mean zero, so, to complete the proof of central limit theorem, we need to calculate the corresponding variance. To compute the covariance matrix $\mathrm{Cov}(V(\tau_{\infty }),V(\tau_{\infty }))$, we use Eq. (2.21) from [44], ch. 10 which translates to

3.15 $$\mathrm{Cov}(V(t),V(t))=\int_{0}^{t}\Phi (t,s)G(x(s),y(s)){\left[\Phi (t,s)\right]}^{T}\;ds.$$

In our case,

$$G(x,y)=\left(\begin{array}{cc} \lambda x & -\lambda x\\ -\lambda x & (\lambda -\alpha )x-\alpha y+\alpha \end{array}\right)$$

and

$$\Phi (t,s)=\left(\begin{array}{cc} e^{-\lambda (t-s)} & 0\\ e^{\alpha (t-s)}-e^{-\lambda (t-s)} & e^{\alpha (t-s)} \end{array}\right),$$

thus we obtain that $\mathrm{Cov}(V(\tau_{\infty }),V(\tau_{\infty }))$ is given by

$$\left(\begin{array}{cc} x_{\infty }-\frac{x_{\infty }^{2}}{x_{0}} & \frac{x_{\infty }(x_{\infty }-x_{0})}{x_{0}}\\ \frac{x_{\infty }(x_{\infty }-x_{0})}{x_{0}} & 2x_{\infty }-1+\frac{{\left(x_{\infty }-1\right)}^{2}}{z_{0}}-\frac{x_{\infty }^{2}}{x_{0}} \end{array}\right).$$

We get the closed formula (3.13) for the asymptotic variance by using last expression and properties of variance.

As mentioned previously, Kortchemski [18] deals with this model on the complete graph in the context of epidemic spreading. More precisely, the case *X*(0)=*n* and *Y* (0)=*Z*(0)=1 is considered in a population of size *n*+2. Interesting results related to limit theorems and phase transitions are obtained. The results stated here concerning the asymptotic behaviour of the rumour process are proved under a different initial configuration and have a different convergence scale. We observe that the case considered in [18] is, using our notation, *x*_0_=1 and *y*_0_=*z*_0_=0 (see equation (3.1)). Therefore, our work complements the results by Kortchemski [18].

## 4. Heterogeneously mixing populations

As an interacting particle system, our model can be formulated in a finite graph (or network) *G* as a continuous-time Markov process (*η*_*t*_)_*t*≥0_ on the state space {0,1,2}^*V*^, where *V* :={1,2,…,*n*} is the set of nodes. A state of the process is a vector *η*=(*η*(*i*):*i*∈*V*), where *η*(*i*)∈{0,1,2} and 0, 1, 2 represent the ignorant, spreader and stifler states, respectively. The rumour is spread at rate λ and a spreader becomes a stifler at rate *α* after contacting stiflers. We assume that the state of the process at time *t* is *η* and let *i*∈*V* . Then

$$P(\eta_{t+h}(i)=1|\eta_{t}(i)=0)=\lambda hN_{1}(i)+o(h)$$

and

$$P(\eta_{t+h}(i)=2|\eta_{t}(i)=1)=\alpha hN_{2}(i)+o(h),$$

where *N*_ℓ_(*i*):=*N*_ℓ_(*η*,*i*) is the number of neighbours of *i* that are in state ℓ, for ℓ=1,2 and for the configuration *η*. In the previous section, we present a rigorous analysis of our rumour model on a complete graph with *n* vertices. Our results in such a case are related to the asymptotic behaviour of the random variables

$$X^{\left(n\right)}(t)=\sum_{i=1}^{n}I_{\left\{\eta_{t}(i)=0\right\}}$$

and

$$Y^{\left(n\right)}(t)=\sum_{i=1}^{n}I_{\left\{\eta_{t}(i)=1\right\}},$$

where *I*_*A*_ denotes the indicator random variable of the event *A*. This mean-field approximation assumes that the possible contacts between each pair of individuals occur with the same probability. This assumption enables an analytical treatment, but does not represent the organization of real-world networks, whose topology is very heterogeneous [39,46–48]. In this case, we use a different approach that allows us to describe the evolution of each node. Such formulation assumes the independence among the state of the nodes. More precisely, we are interested in the behaviour of the probabilities

4.1 $$\begin{array}{rl} x_{i}(t) & :=P(\eta_{t}(i)=0),\\ y_{i}(t) & :=P(\eta_{t}(i)=1)\\ \;\text{and}\;\;z_{i}(t) & :=P(\eta_{t}(i)=2), \end{array}\}$$

for all *i*=1,2,…,*n*. We describe our process in terms of a collection of independent Poisson processes $N_{i}^{\lambda }$ and $N_{i}^{\alpha }$ with intensities λ and *α*, respectively, for *i*=1,2,…,*n*. We associate the processes $N_{i}^{\lambda }$ and $N_{i}^{\alpha }$ to the node *i* and we say that at each time of $N_{i}^{\lambda }$ ($N_{i}^{\alpha }$), if *i* is in state 1 (2) then it chooses a nearest neighbour *j* at random and tries to transmit (scotch) the information provided *j* is in state 0 (1). In this way, we obtain a realization of our process (*η*_*t*_)_*t*≥0_.

In order to study the evolution of the functions (4.1), we fix a node *i* and analyse the behaviour of its different transition probabilities on a small-time window. More precisely, consider a small enough positive number *h* and note that

4.2 $$P(\eta_{t+h}(i)=0)=P(\eta_{t+h}(i)=0|\eta_{t}(i)=0)P(\eta_{t}(i)=0),$$

where the first factor of the right-hand side of last expression is given by

4.3 $$\begin{array}{rl} P(\eta_{t+h}(i)=0|\eta_{t}(i)=0) & =1-P(\eta_{t+h}(i)=1|\eta_{t}(i)=0)-P(\eta_{t+h}(i)=2|\eta_{t}(i)=0)\\ & =1-P(\eta_{t+h}(i)=1|\eta_{t}(i)=0)+o(h). \end{array}$$

The *o*(*h*) term appears in the above equation, because the occurrence of a transition from state 0 to state 2 in a time interval of size *h* implies the existence of at least two marks of a Poisson process at the same time interval.

To develop (4.3), for a node *j*, let $B_{ji}(h)$ denote the intersection of the events: (i) {*N*_*j*_(*t*,*t*+*h*)=1}; (ii) {*j* transmit the information to *i* *in* (*t*,*t*+*h*)}; (iii) {*η*_*j*_(*t*)=1}; and (iv) {*η*_*j*_(*s*)=1, *for* *t*<*s*≤*t*+*h*}. Also let *A*_*ji*_=1 if *i* is a direct neighbour of *j* in the network (equals 0 other case) and $k_{i}=\sum_{j}A_{ij}$ is the degree of the node *i*.

We observe that the event (i) only takes into account the Poisson process with rate λ, and that the probability of a contact between nodes *j* and *i*, which is related to (ii), is given by *A*_*ji*_/*k*_*j*_.

Consequently, we obtain

4.4 $$\begin{array}{rl} P(\eta_{t+h}(i)=1|\eta_{t}(i)=0) & =P(\eta_{t+h}(i)=1|\eta_{t}(i)=0,\cup_{j=1}^{n}B_{ji}(h))P(B_{ji}(h)|\eta_{t}(i)=0)+o(h),\\ & =\sum_{j=1}^{n}\frac{A_{ji}}{k_{j}}(\lambda h+o(h))P(\eta_{t}(j)=1)+o(h). \end{array}$$

Thus, we obtain

$$P(\eta_{t+h}(i)=0)=\left(1-\sum_{j=1}^{n}\frac{A_{ji}}{k_{j}}(\lambda h+o(h))P(\eta_{t}(j)=1)+o(h)\right)P(\eta_{t}(i)=0)$$

or

$$P(\eta_{t+h}(i)=0)-P(\eta_{t}(i)=0)=-\left(\sum_{j=1}^{n}\frac{A_{ji}}{k_{j}}(\lambda h+o(h))P(\eta_{t}(j)=1)+o(h)\right)P(\eta_{t}(i)=0).$$

Finally, as $x_{i}^{\prime }(t)=\lim_{h\to 0}(x_{i}(t+h)-x_{i}(t))/h$ we conclude $x_{i}^{\prime }(t)=-\lambda x_{i}(t)\sum_{j=1}^{n}(A_{ji}/k_{j})y_{j}(t).$ Same arguments allow us to obtain the equations for *y*_*i*_(*t*) and *z*_*i*_(*t*). In this way, we have the following set of dynamical equations:

4.5 $$\begin{array}{rl} x_{i}^{\prime }(t) & =-\lambda x_{i}(t)\sum_{j=1}^{n}P_{ji}y_{j}(t),\\ y_{i}^{\prime }(t) & =\lambda x_{i}(t)\sum_{j=1}^{n}P_{ji}y_{j}(t)-\alpha y_{i}(t)\sum_{j=1}^{n}P_{ji}z_{j}(t),\\ z_{i}^{\prime }(t) & =\alpha y_{i}(t)\sum_{j=1}^{n}P_{ji}z_{j}(t)\\ \;\text{and}\;\;x_{i}(0) & =x_{0},\;y_{i}(0)=y_{0},\;z_{i}(0)=z_{0}, \end{array}\}$$

for all *i*=1,2,…,*n*, and *P*_*ji*_:=*A*_*ji*_/*k*_*j*_. We observe that when the network considered is a complete graph of *n* vertices, the system of equations (4.5) matches with the homogeneous approach (see the system of equations (3.2)).

Observe that our formalism assumes that the network is fixed and static during the whole spreading process. Such formalism is similar to the so-called quenched mean field (QMF) for epidemic spreading [49,50]. In this manner, for a fixed network we have one set of equations that describes its behaviour. Such approach contrasts with the heterogeneous mean field (HMF), applied to epidemic spreading [51,52] and the MT in [40,52]. Regarding the HMF, only the degree distribution is considered and all nodes with degree *k* are considered statistically equivalent. Such formalism neglects specific structures of the network (e.g. the number of triangles), as many different networks can have the same degree distribution.

In order to verify the influence of network structure on the dynamical behaviour of the models, we consider random graphs of ER and scale-free networks of Barabási and Albert (BA). Random graphs are created by a Bernoulli process, connecting each pair of vertices with the same probability *p*. The degree distribution of random graphs follows a Poisson distribution for large values of *n* and small *p*, as a consequence of the law of rare events [53]. On the other hand, the BA model generates scale-free networks by taking into account the network growth and preferential attachment rules [54]. The networks generated by this model present degree distribution following a power-law, *P*(*k*)∼*k*^−*γ*^, with *γ*=3. In random graphs most of the nodes have similar degrees, whereas scale-free networks are characterized by a very heterogeneous structure.

**Figure 4.**
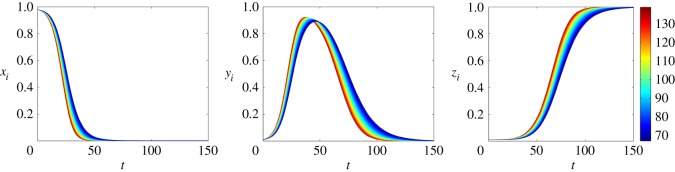
Time evolution of the nodal probabilities considering our model for an ER network with *n*=10^4^ nodes and 〈*k*〉≈100. We consider the spreading rate λ=0.2 and stifling rate *α*=0.1. Each curve represents the probability that a node is in one of the three states (ignorant, spreader or stifler) and the colour represents the degree of the node *i*. The initial conditions are *x*_0_=0.98, *y*_0_=0.01 and *z*_0_=0.01.

**Figure 5.**
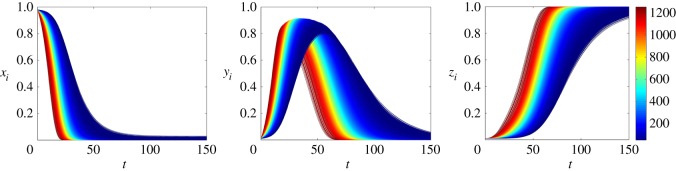
Time evolution of the nodal probabilities considering our model for an BA network with *n*=10^4^ nodes and 〈*k*〉≈100. The spreading rate as λ=0.2, while the stifling rate is *α*=0.1. Each curve represents the probability that a node is in one of the three states (ignorant, spreader or stifler) and the colour represents the degree of the node *i*. The initial conditions are *x*_0_=0.98, *y*_0_=0.01 and *z*_0_=0.01.

Figures 4 and 5 show the time evolution of the nodal probabilities, considering ER and BA networks, respectively. These results are obtained by solving numerically the system of equations (4.5). Both networks have *n*=10^4^ nodes and 〈*k*〉≈100. The spreading rate is λ=0.2 and the stifling rate is *α*=0.1. The colour of each curve denotes the degree of each node *i*. Comparing figures 4 and 5, we can see that the variance of *x*_*i*_,*y*_*i*_ and *z*_*i*_ in BA networks is higher than in ER networks. Moreover, in both networks, higher degree nodes tend to turn into a stifler earlier than lower degree ones.

**Figure 6.**
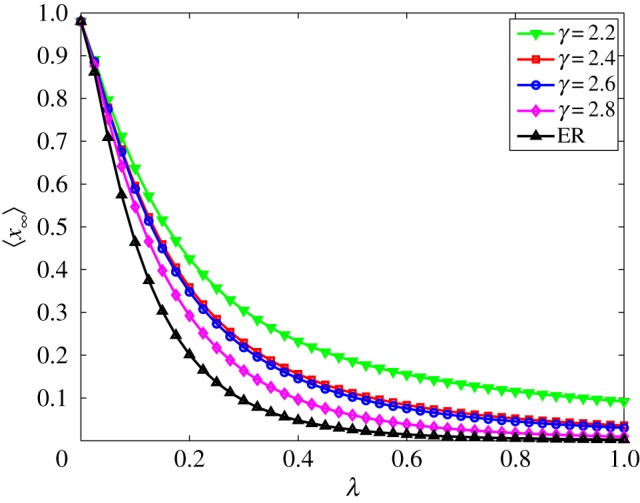
Phase diagram of the final fraction of ignorants as a function of λ for *α*=0.5, $\langle x_{\infty }\rangle \times \lambda$. The initial conditions are *x*_0_=0.98, *y*_0_=0.01 and *z*_0_=0.01. All the networks have *n*=10^3^ nodes and 〈*k*〉≈10.

In addition to the homogeneous versus heterogeneous comparison performed before, we can also compare different levels of heterogeneity. As many real networks rely on power-law degree distributions, *P*(*k*)∼*k*^−*γ*^, with 2<*γ*<3 [55] we use the configuration model [56] to generate such networks without degree correlations. More precisely, we use the algorithm proposed in [57]. Figure 6 shows the phase diagram of the final fraction of ignorants as a function of λ for *α*=0.5. Here we use five networks with *n*=10^3^ nodes and 〈*k*〉≈10, four of them are power-law degree distributions, *P*(*k*)∼*k*^−*γ*^ with *γ*=2.2, *γ*=2.4, *γ*=2.6 and *γ*=2.8 and one ER. Besides the initial conditions are *x*_0_=0.98, *y*_0_=0.01 and *z*_0_=0.01. This experiment is based on the numerical solution of the ODE set of equations (4.5) for a sufficiently large value of time, where the number of spreaders is negligible. We observe that the higher the *γ* the lower the final fraction of ignorants, suggesting that the spreading is favoured by such structural feature. Interestingly, the ER network showed the lowest final fraction of ignorants. Regarding scale-free networks, such results suggest that hubs on our model present a similar behaviour to hubs on the MT model, suggesting that on a first moment, hubs favour the spreading; however, once it becomes a stifler it acts efficiently, stifling its neighbours. Again, similarly to MT model, the homogeneity seems to favour the spreading which contrasts with the epidemic spreading processes, which are favoured by heterogeneity.

We compare the behaviour of our model, described by equation (4.5), with the MT model [24] in ER and BA networks. The time evolution of this model is given by

4.6 $$\begin{array}{rl} x_{i}^{\prime }(t) & =-\lambda x_{i}(t)\sum_{j=1}^{n}P_{ji}y_{j}(t),\\ y_{i}^{\prime }(t) & =\lambda x_{i}(t)\sum_{j=1}^{n}P_{ji}y_{j}(t)-\alpha y_{i}(t)\sum_{j=1}^{n}P_{ij}(x_{j}(t)+z_{j}(t)),\\ z_{i}^{\prime }(t) & =\alpha y_{i}(t)\sum_{j=1}^{n}P_{ij}(x_{j}(t)+z_{j}(t))\\ \;\text{and}\;\;x_{i}(0) & =x_{0},\;y_{i}(0)=y_{0},\;z_{i}(0)=z_{0}, \end{array}\}$$

where, as before, *x*_*i*_, *y*_*i*_ and *z*_*i*_ are the micro-state variables, quantifying the probability that the node *i* is an ignorant, a spreader or a stifler at time *t*, respectively, for *i*=1,2,…,*n*. Note *x*_*i*_(*t*)+*y*_*i*_(*t*)+*z*_*i*_(*t*)=1,∀*i*,*t*.

**Figure 7.**
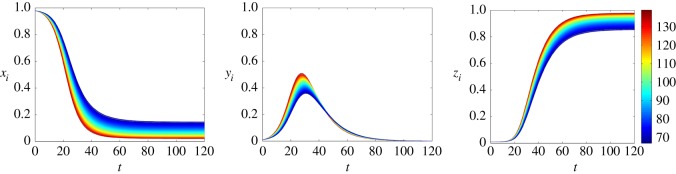
Time evolution of the nodal probabilities considering the MT model in an ER network with *n*=10^4^ nodes and 〈*k*〉≈100. The spreading rate is λ=0.2 and the stifling rate is *α*=0.1. Each curve represents the probability that a node is in one of the three states (ignorant, spreader or stifler) and the colour represents the degree of the node *i*. The initial conditions are *x*_0_=0.98, *y*_0_=0.01 and *z*_0_=0.01.

**Figure 8.**
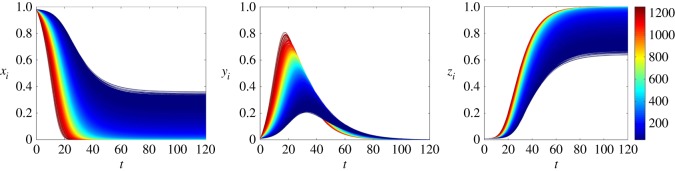
Time evolution of the nodal probabilities considering the MT model in an BA network with *n*=10^4^ nodes and 〈*k*〉≈100. The spreading rate is λ=0.2 and the stifling rate is *α*=0.1. Each curve represents the probability that a node is in one of the three states (ignorant, spreader or stifler) and the colour represents the degree of the node *i*. The initial conditions are *x*_0_=0.98, *y*_0_=0.01 and *z*_0_=0.01.

Figures 7 and 8 show the time evolution of the nodal probabilities, by numerically solving equation (4.6). Similarly to our model, the variances in BA networks are higher than in ER networks. Besides, the hubs and leaves of the BA networks present a completely different behaviour, as can be seen in figure 8*b*. Moreover, the nodes having higher degrees also tend to become stifler earlier than low degree nodes.

We consider the same initial conditions for both rumour models, i.e. *x*_0_=0.98, *y*_0_=0.01 and *z*_0_=0.01. It is worth emphasizing that the initial conditions in figures 7 and 8 are not usual in the MT model, as most of the works on this model considers the initial fraction of stiflers as zero [1]. However, our model needs an initial non-zero fraction of stiflers, otherwise there is no manner to contain the rumour propagation, so we assumed the same initial condition in order to perform a comparative analysis of both models, as important differences emerge. The main feature that emerges from the comparison between figures 5 and 4 with 8 and 7 is the peak of the probability of a node being a spreader. In our model it tends to be higher than in the MT process. Such a feature evinces the differences between two formulations. In the MT model, the spreaders lose the interest in the rumour propagation owing to the contact with individuals who have already known the rumour, whereas in our model spreaders are convinced only by stifler vertices to stop spreading the information.

As mentioned before, the hubs on our model present a similar behaviour as on the MT model, having a large number of edges it spreads and is stifled very efficiently. Aside from this similarity on the MT such phenomenon happens at a faster rate, as an individual can lose interest on the rumour just by contacting twice to one of its neighbour individuals (on the first contact spreads the information, on the second it becomes a stifler, subject to the rates of the process). On the other hand, in our model stiflers are active and depend only on the probability of finding a spreader. In this manner, at the beginning of the process *y*_*i*_ and *z*_*i*_ are low, implying that $z_{i}^{\prime }(t)$ is also low and the dominant term of $y_{i}^{\prime }(t)$ is $\lambda x_{i}(t)\sum_{j=1}^{n}P_{ji}y_{j}(t)$. Then, when the fraction of spreaders increase it also increases the $z_{i}^{\prime }(t)$. Such a process seems to be faster on the MT model than ours. Consequently, the final fraction of ignorants and the time to achieve the steady state are different.

In addition, it is noteworthy that the MT model allows an individual to spread the information to a neighbour, then lost interest by contacting the same individual, which seems to be different from real-world situations. Such feature is absent in our model, however, in our model the individuals do not lose interest in the information, they are convinced to stop the spreading.

## 5. Monte Carlo simulation

The analytical methods presented in §§3 and 4 assume that there is no correlation between the states. However, it is not true on most real cases, due to triangles, assortativity, community structure, among other features. This is also the assumption made on many epidemic [49–52] and rumour spreading models [40,52]. In [58], the authors compared the accuracy of some mean field approaches, considering many different dynamical processes, and showed that some approaches present relative accuracy on disassortative networks even when the mean degree is low. Although some approximations are still valid, the Monte Carlo simulations mimic the process itself in a computational manner assuming only the pattern of connections and the contact relationships. As it does not assume the absence of correlations, those simulations are expected to be more similar to the real process. In this manner, the analytical and numerical methods exposed in §4 and the Monte Carlo simulations are complementary. On the one hand, the ODE system give us insights about the process, allowing us to threaten it mathematically, on the other hand, the Monte Carlo simulations make less assumptions.

In this way, we perform extensive numerical simulations to verify how our rigorous results obtained for homogeneously mixing populations can be considered as approximations for random graphs and scale-free networks. The rumour spreading simulation is based on the contact between two individuals. At each time step each spreader makes a trial to spread the rumour to one of its neighbours and each stifler makes a trial to stop the spreading. If the spreader contacts an ignorant, it spreads the rumour with probability λ. Similarly, if the stifler contacts an spreader that spreader becomes a stifler with probability *α*. The updates are performed in a sequential asynchronous fashion. For the simulation procedure, it is important to randomize the state of the initial conditions, especially for the heterogeneous networks. In order to overcome statistical fluctuations in our simulations, every model is simulated 50 times with random initial conditions.

### 5.1 Complete graph

**Figure 9.**
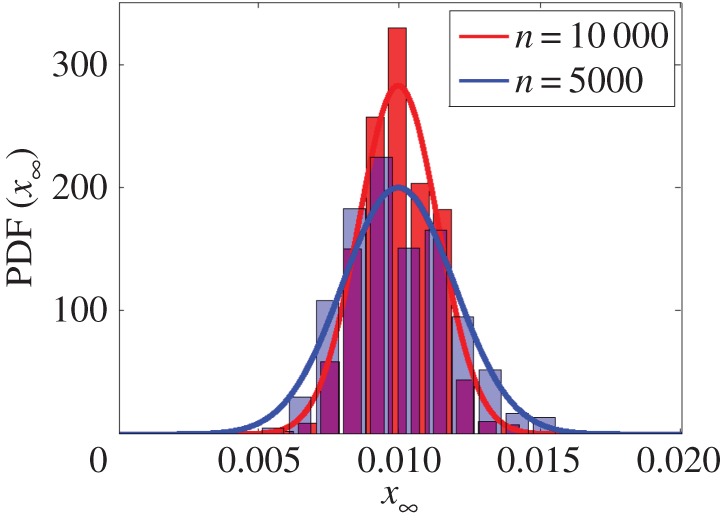
Distribution of the fraction of ignorants obtained from 1000 simulations in a complete graph varying the number of nodes. The bars are obtained experimentally, while the fitted Gaussian are based on the theoretical values obtained from equations (3.2), (3.8) and (3.12).

The results are quantified as a function of the fraction of ignorant nodes, as when the time tends to infinity, the proportion of spreaders tends to zero and the fraction of ignorants and stiflers has complementary information about the population. Figure 9 compares the distribution of the fraction of ignorants obtained by Monte Carlo simulations with the central limit theorem by fitting a Gaussian distribution according to the theoretical values obtained from equations (3.2), (3.8) and (3.12). Complete graphs of two different sizes are considered to show the dependency on the number of nodes *n*. Note that equations (3.8) and (3.12) assert that only the variance depends on the network size, i.e. $\sigma^{2}\propto 1/\sqrt{n}$. Thus, the numerical simulations agree remarkably with the theoretical results.

### 5.2 Complex networks

**Figure 10.**
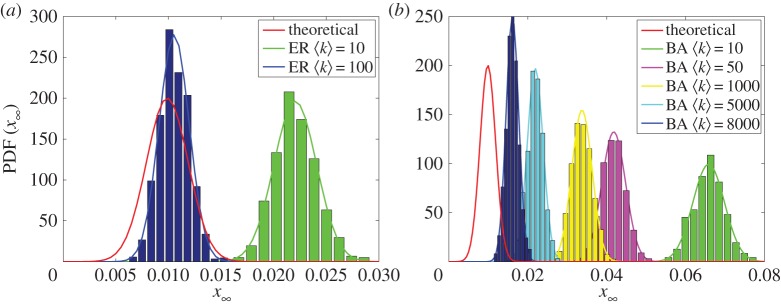
Distribution of the fraction of ignorants considering 1000 Monte Carlo simulations of the rumour scotching model in networks with *n*=10^4^ nodes generated from the (*a*) ER and (*b*) BA network models. The simulations consider λ=0.5, *α*=0.5 and initial conditions *x*_0_=0.98, *y*_0_=0.01 and *z*_0_=0.01. Theoretical curves, obtained by equations (3.2), (3.8) and (3.12), are in red.

In order to verify the behaviour of the rumour scotching model on complex networks, we evaluate networks generated by random graphs of the ER and scale-free networks of BA. Figure 10 shows the distribution of the final fraction of ignorants considering 1000 Monte Carlo simulations of the rumour scotching model in networks with *n*=10^4^ vertices generated from the ER and BA models. The theoretical results for the homogeneously mixing populations, obtained from equations (3.2), (3.8) and (3.12), are also shown. In ER networks, the distribution converges to the theoretical results as the network becomes denser. In this way, even in sparse networks, 〈*k*〉=100, the results are close to the mean-field predictions. On the other hand, the convergence of scale-free networks to the theoretical results does not occur even for 〈*k*〉=8000 because of their high level of heterogeneity.

The system of equations (3.2) that describes the evolution of rumour dynamics on homogeneous populations can characterize the same dynamics in random regular networks if we consider λ=〈*k*〉λ′ and *α*=〈*k*〉*α*′. In this case, the probabilities of spreading and scotching the rumour depend on the number of connections, but the solution of the system of equations does not change. As random networks present an exponential decay near the mean degree, their dynamical behaviour is similar to the mean-field predictions. On the other hand, this approximation is not accurate for scale-free networks, because they do not present a typical degree and the second-moment of their degree distribution diverges for 2<*γ*≤3 as n→∞. Therefore, the homogeneous mixing assumption is suitable only for ER networks.

**Figure 11.**
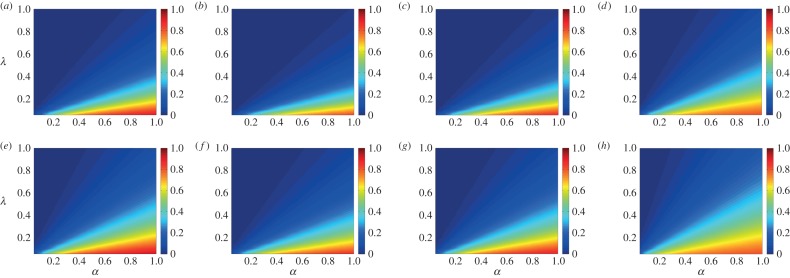
Fraction of ignorants (given by colour intensities) according to the rates *α* and λ for different initial conditions considering ER, from (*a*–*d*), and BA network models, from (*e*–*h*). Networks with *n*=10^4^ and 〈*k*〉≈8 are considered. Every point is as an average over 50 simulations. (*a*) *x*_0_=0.98, *y*_0_=0.005 and *z*_0_=0.015, (*b*) *x*_0_=0.98, *y*_0_=0.015 and *z*_0_=0.005, (*c*) *x*_0_=0.98, *y*_0_=0.01 and *z*_0_=0.01, (*d*) *x*_0_=0.9, *y*_0_=0.05 and *z*_0_=0.05, (*e*) *x*_0_=0.98, *y*_0_=0.005 and *z*_0_=0.015, (*f*) *x*_0_=0.98, *y*_0_=0.015 and *z*_0_=0.005, (*g*) *x*_0_=0.98, *y*_0_=0.01 and *z*_0_=0.01, and (*h*) *x*_0_=0.9, *y*_0_=0.05 and *z*_0_=0.05.

Figure 11 shows the Monte Carlo simulation results as a function of the parameters *α* and λ for different initial conditions. The simulation considers every pair of parameters, λ and *α*, starting from λ=*α*=0.05 and incrementing them with steps of 0.05 until reaching the unity. In the rumour spreading dynamics, the role played by the stiflers is completely different from the recovered individuals in epidemic spreading. Note that stifler and recovered are absorbing states. However, in the disease spreading, the recovered individuals do not participate in the dynamics and are completely excluded from the interactions, whereas in our model, stiflers are active and try to scotch the rumour to the spreaders.

The number of connections of the initial propagators influences the spread of disease [21,59], but does not impact the rumour dynamics [60]. We investigate if the number of connections of the initial set of spreaders and stiflers affects the evolution of the rumour process with scotching in BA scale-free networks. In a first configuration, the initial state of the hubs is set as spreaders and stiflers are distributed uniformly in the remaining of the network. In another case, stiflers are the main hubs and spreaders are distributed uniformly. In both cases, we verify that the final fraction of ignorants is the same as in completely uniform distribution of spreader and stifler states (figure 11*e*–*h*). Therefore, we infer that the degree of the initial spreaders and stiflers does not influence the final fraction of ignorants.

**Figure 12.**
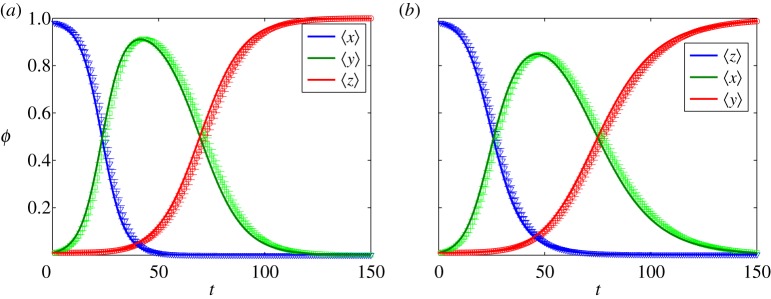
Comparison of the Monte Carlo simulations and the solution of the nodal time evolution differential equations, equations (4.5). The continuous curves are the numerical solution of the differential equations (4.5), while the symbols are the Monte Carlo simulations with its respective standard deviation. Every point is as an average over 50 simulations. In (*a*) an ER network while in (*b*) a BA network. Both with *n*=10^4^ nodes and 〈*k*〉≈100. Moreover, the initial conditions are *x*_0_=0.98, *y*_0_=0.01 and *z*_0_=0.01.

Figure 12 shows numerical solutions of equation (4.5) and the Monte Carlo simulations for ER and BA networks. Regarding the simulations, figure 12*a*,*b* correspond to the average behaviour of the variables shown in figures 4 and 5. We can see that the maximum fraction of spreaders occurring in BA networks is lower than in ER networks. This happens because most of the vertices in BA networks are lowly connected (owing to the power-law degree distribution). Moreover, we can see that the variance decays over time, which is a consequence of the presence of an absorbing state. In addition, we also find that for sparser networks the matching is less accurate (results not shown).

## 6. Conclusion

The modelling of rumour-like mechanisms is fundamental to understanding many phenomena in society and online communities, such as viral marketing or social unrest. Many works have investigated the dynamics of rumour propagation in complete graphs (e.g. [22]) and complex structures (e.g. [40]). The models considered so far assume that spreaders try to propagate the information, whereas stiflers are not active. Here, we propose a new model in which stiflers try to scotch the rumour to the spreader agents. We develop an analytical treatment to determine how the fraction of ignorants behaves asymptotically in finite populations by taking into account the homogeneous mixing assumption. We perform Monte Carlo simulations of the stochastic model on ER random graphs and BA scale-free networks. The results obtained for homogeneously mixing populations can be used to approximate the case of random networks, but are not suitable for scale-free networks, owing to their highly heterogeneous organization. The influence of the number of connections of the initial spreaders and stiflers is also addressed. We verify that the choice of hubs as spreaders or stiflers has no influence on the final fraction of ignorants.

The study performed here can be extended by considering additional network models, such as small-world or spatial networks. The influence of network properties, such as assortativity and community organization can also be analysed in our model. In addition, strategies to maximize the range of the rumour when the scotching is present can also be developed. The influence of the fraction of stiflers on the final fraction of ignorant vertices is another property that deserves to be investigated.
